# Healthcare Interventions in the Management of Rheumatic Diseases: A Narrative Analysis of Effectiveness and Emerging Strategies

**DOI:** 10.3390/healthcare13141691

**Published:** 2025-07-14

**Authors:** Gabriela Isabela Verga (Răuță), Alexia Anastasia Ștefania Baltă, Diana-Andreea Ciortea, Carmen Loredana Petrea (Cliveți), Mariana Șerban (Grădinaru), Mădălina Nicoleta Matei, Gabriela Gurău, Victoria-Cristina Șuța, Doina Carina Voinescu

**Affiliations:** 1 Faculty of Medicine and Pharmacy, University “Dunărea de Jos” of Galati, 800008 Galati, Romania; alexiabalta@yahoo.com (A.A.Ș.B.); carmen.petrea@ugal.ro (C.L.P.); madalina.matei@ugal.ro (M.N.M.); gabriela.gurau@ugal.ro (G.G.); doina.voinescu@ugal.ro (D.C.V.); 2 Emergency Clinical Hospital for Children “Sf Ioan”, 800487 Galati, Romania; mariana_gradinaru@yahoo.com; 3 St. “Apostol Andrei” County Emergency Clinical Hospital, 800578 Galati, Romania; 4 Emergency Clinical Hospital for Children “Maria Sklodowska Curie”, 041451 Bucharest, Romania; 5 Faculty of Medicine, University Ovidius of Constanta, 900470 Constanta, Romania; cris_duminica@yahoo.com; 6 St. “Apostol Andrei” County Emergency Clinical Hospital, 900591 Constanta, Romania

**Keywords:** nursing interventions, rheumatic diseases, pain management, patient education, multidisciplinary care, functional rehabilitation, healthcare accessibility

## Abstract

Background and aims: Rheumatic diseases are chronic, progressive conditions associated with severe pain, joint damage, disability, and even death. Healthcare interventions play a critical role in symptom management, patient education, and adherence to treatment plans. This study evaluates the role of healthcare interventions in the management of patients with rheumatic diseases, focusing on pain management, functional rehabilitation, patient education, and multidisciplinary collaboration. In addition, barriers to optimal care and potential solutions, including digital health technologies, are explored. Materials and methods: We conducted a narrative review of the scientific literature. Studies published between 2014 and 2025 were selected from PubMed, Scopus, Web of Science, Elsevier, Springer, Frontiers, and Wiley Online Library. Key areas of review included nurse-led pain management, education programs, and the impact of interdisciplinary care on patient outcomes. Results: Nursing interventions significantly improve pain control, treatment adherence, and self-management skills in patients with rheumatic diseases. Multidisciplinary approaches improve functional rehabilitation and increase quality of life in patients with rheumatic conditions. However, barriers such as insufficient health care resources, lack of patient awareness, and disparities in the availability of services hinder effective care delivery. Conclusions: A structured, multidisciplinary approach integrating healthcare interventions, digital health solutions, and patient-centered education is essential to optimize the management of rheumatic diseases. Future research should focus on improving access to non-pharmacological therapies and standardizing healthcare protocols for better patient outcomes.

## 1. Introduction

Rheumatic diseases encompass a wide range of chronic musculoskeletal and systemic conditions, including both inflammatory conditions such as rheumatoid arthritis (RA), systemic lupus erythematosus (SLE), and spondyloarthritis, and degenerative diseases such as osteoarthritis [1,2,3,4,5]. Overall, these conditions represent a significant global burden, contributing to long-term disability, reduced quality of life, and increased healthcare costs [6,7,8,9].

While inflammatory rheumatic diseases involve immune-mediated synovial inflammation and systemic organ damage, non-inflammatory forms are driven by biomechanical stress and metabolic alterations. Regardless of etiology, patients often present with chronic pain, joint stiffness, fatigue, and functional limitations [10,11,12,13,14].

Disease progression is influenced by multiple factors, including genetic predisposition, environmental triggers (e.g., smoking, infections), metabolic status (e.g., obesity, insulin resistance), and behavioral patterns (e.g., physical inactivity, diet) [15,16,17,18,19,20]. In addition, systemic inflammation in rheumatic diseases has been linked to increased cardiovascular morbidity, endothelial dysfunction, and accelerated atherosclerosis [21,22,23,24,25].

Advances in pharmacologic therapy—such as DMARDs, biologic drugs (e.g., TNF-α and IL-6 inhibitors), corticosteroids, and JAK inhibitors—have improved disease control and patient survival [26,27,28,29,30]. However, optimal long-term management requires an integrated, patient-centered approach that takes into account clinical symptoms and psychosocial well-being. Under the circumstances, the role of the nurse has gained increasing recognition [31,32,33,34].

Nurses are uniquely positioned to provide ongoing support in the care of rheumatic diseases through structured education, promotion of self-management, pain control, monitoring adherence to treatment, and early detection of complications [35,36,37,38,39]. Evidence from observational studies and clinical trials suggests that nursing interventions improve patient-reported outcomes, physical function, and mental health while reducing disease activity and comorbidity risks [40,41,42,43,44,45,46].

The EULAR (European Alliance of Associations for Rheumatology) recommendations have emphasized the importance of multidisciplinary care and identified nursing as the cornerstone of effective management [47,48,49,50]. However, a consistent gap persists between the guidelines and their implementation in clinical practice, in part due to variation in training, institutional support, and lack of standardized protocols for nursing interventions [51,52,53,54].

In addition, rheumatic diseases often coexist with metabolic and micronutrient imbalances (e.g., vitamin D, folic acid, B12), which can exacerbate disease severity. Healthcare interventions incorporating dietary assessment, supplementation guidance, and interdisciplinary referral have shown promising results in mitigating these risks [55,56,57,58,59].

Additional factors—such as gut microbiota dysbiosis, systemic inflammation, and the role of extracellular vesicles—have emerged as contributors to both rheumatic pathology and cardiovascular complications [60,61,62,63]. These findings highlight the need for holistic care strategies that prioritize systemic health beyond joint symptoms.

In 2018, the EULAR recommendations were updated, emphasizing the role of the nurse as part of the healthcare team. The general principles support shared decision-making with the patient and describe the importance of providing evidence-based care [64]. The EULAR 2018 recommendations highlight the need to improve nurses’ practice and knowledge in terms of education, clinical skills, and work organization. To prevent the risk of a high workload that could influence the quality of care, the appropriate organization of care by nurses is fundamental [65,66,67].

In the last 10 years, the care of patients with rheumatic diseases has changed considerably due to the early initiation of treatment and close monitoring of disease activity until remission is achieved. Individualized approach to patients with rheumatic diseases provides a sense of security due to the broad spectrum of clinical symptoms, which affect patients’ quality of life [68,69,70].

The primary objective of this narrative review was to explore how health care interventions influence disease outcomes and quality of life in patients with rheumatic diseases, with a particular emphasis on comparing the effectiveness of these interventions and identifying promising alternatives for improving clinical outcomes and quality of life [71,72,73].

The review aimed to identify key areas in which healthcare actions improve patient outcomes and to highlight gaps or inconsistencies in existing practices [74,75]. In addition, the authors evaluated the role of nurses in multidisciplinary care teams, analyzing how interprofessional collaboration improves the management of patients with rheumatic conditions [76,77].

Another key objective was to explore how nursing approaches vary according to the type and severity of rheumatic disease and patient-specific factors [78,79].

The review also addressed barriers to effective care by nurses, including workforce shortages, limited access to specialized professionals, disparities in healthcare infrastructure, and patient-related challenges in adhering to treatment. Finally, the study examined the integration of emerging healthcare strategies such as digital health tools and patient education models [80,81].

## 2. Materials and Methods

This review was conducted as a narrative review, combining the methodological rigor of a systematic review with the narrative approach of synthesis. This method was chosen to allow a detailed exploration of a complex and interdisciplinary topic, including medical care, management of rheumatic patients, and multidisciplinary collaboration in rheumatology. The synthesis of results emphasizes not only the reported data but also the clinical implications, gaps in knowledge, and future research directions.

The literature was selected through a non-systematic search of databases PubMed, Scopus, Web of Science, Elsevier, Springer, Frontiers, and Wiley Online Library, covering the period from January 2014 to January 2025. Keywords such as “nursing interventions, rheumatic diseases, pain management, patient education, multidisciplinary care, functional rehabilitation, healthcare accessibility” were used. Inclusion criteria were peer-reviewed articles in English that focused on the role of nurses in managing rheumatic conditions. Editorials and articles unrelated to clinical nursing interventions were excluded.

### 2.1. Identification of the Research Problem

The central research question: How do nursing interventions influence the disease progression and quality of life of patients with rheumatic diseases, and which strategies are most effective for optimizing patient care?

The study examines nursing interventions and patient outcomes in the management of rheumatic diseases, comparing different models of care and analyzing their effectiveness in controlling pain, improving physical function, and increasing patient adherence to treatment.

It also assesses current challenges in rheumatology care as well as emerging strategies such as digital health solutions and patient-centered nursing models.

The review integrates multiple perspectives to improve patient understanding and management, including:

- Evaluating the effectiveness of non-pharmacologic interventions such as physiotherapy, patient education, and psychological support.
- The role of multidisciplinary collaboration, with regard to the interaction between nurses, rheumatologists, and rehabilitation specialists in developing personalized care plans.
- The impact of nursing interventions on patient outcomes, highlighting how early assessments by nurses contribute to long-term disease management, the effectiveness of structured rehabilitation programs, and the role of ongoing patient education in preventing complications.

The review examines barriers in providing optimal care, such as limited access to specialized nurses, disparities in health system infrastructure, and patient-related factors that influence adherence to care plans.

Gaps in existing knowledge and challenges in integrating nursing interventions into routine rheumatologic practice are identified.

Future research directions are proposed for optimizing nursing strategies, improving interdisciplinary collaboration, and enhancing the quality of patient-centered care in the management of rheumatic diseases.

### 2.2. Identifying Relevant Articles

In order to answer the research questions, a search was performed in the following databases: PubMed, Scopus, Web of Science, Elsevier, Springer, Frontiers, and Wiley Online Library.

The main keywords used in the search were: nursing interventions, rheumatic diseases, pain management, patient education, multidisciplinary care, functional rehabilitation, and healthcare accessibility.

These terms were combined into structured search strings to maximize relevance and select high-quality studies.

Each database was searched using tailored search strategies to account for differences in indexing systems and search functions. In addition to standard keyword searches, the following methods were used:

- Citation tracking—identifying additional relevant studies based on references cited in key articles.
- Related article exploration—using built-in features in databases such as ‘Similar Articles’ in PubMed and the Scopus citation network to find studies closely related to the research topic.

This multi-layered approach provided a comprehensive dataset that included essential studies, reducing the risk of omission of essential research on nursing interventions in rheumatic disease care.

Only full English-language articles were included to ensure access to high-impact international studies published in prestigious peer-reviewed journals.

Only peer-reviewed studies were considered, prioritizing clinical trials evaluating the effectiveness of healthcare interventions in the management of rheumatic diseases, systematic reviews and meta-analyses providing a synthesis of existing research, observational studies examining the impact of healthcare on disease progression and quality of life of patients, qualitative studies exploring patients’ and nurses’ experiences in the management of rheumatic diseases.

Review articles were prioritized if they provided a comprehensive synthesis of existing data, while clinical trials were analyzed for their insights into nurse-led interventions, multidisciplinary care, and patient adherence to treatment plans.

### 2.3. Eligibility Criteria for Study Selection

#### 2.3.1. Inclusion Criteria

The selected articles had to focus on the nursing process in the care of patients with rheumatic diseases, particularly in terms of patient assessment, nursing interventions, and multidisciplinary care strategies. Publications had to provide information on:

- Nursing interventions including pain management, functional rehabilitation, patient education, and psychological support in the care of patients with rheumatic diseases.
- Assessment strategies, such as standardized functional assessment tools, pain scales, and clinical assessment methods, are used by nurses to monitor disease progression.
- Multidisciplinary collaboration that examines the role of nurses in interdisciplinary teams, including rheumatologists, physiotherapists, occupational therapists, and other health professionals.
- Patient adherence and self-care strategies, exploring the impact of nurse-led interventions on improving treatment adherence and quality of life.

The review included original research articles, systematic reviews, meta-analyses, and clinical trials published in indexed, peer-reviewed journals, ensuring the inclusion of high-quality, evidence-based sources.

#### 2.3.2. Exclusion Criteria

The following studies were excluded from the review:

- Case reports that did not have wider applicability or did not provide sufficient data on healthcare interventions and patient outcomes.
- Studies that focused exclusively on pharmacologic treatments without discussing the role of nursing care in the management of rheumatic diseases.
- Articles that did not include original data or were published in non-peer-reviewed sources were excluded, ensuring that only high-quality, scientifically validated research was considered.

#### 2.3.3. Selection Process

To guarantee relevance, methodological rigor, and the caliber of the final selection, all identified papers were assessed using predetermined inclusion and exclusion criteria. The last set of research offers a thorough summary of nursing procedures, encompassing patient evaluation, pain management, functional rehabilitation, and interprofessional teamwork.

The selection procedure consisted of two separate stages:

Phase 1: Screening of titles and abstracts—The titles and abstracts were screened to assess general relevance, with an emphasis on research that examined medical treatment, patient outcomes, and healthcare approaches for treating rheumatic illnesses. At this point, studies that did not specifically address these issues or that lacked real data from their own research were eliminated.

Phase 2: Complete Text Analysis—The remaining studies were subjected to a thorough full-text analysis, with a focus on research that:

- Investigated the links between healthcare treatments and patient outcomes, as well as the significance of multidisciplinary teams in rheumatology treatment;
- Talked about ways to educate patients and help them adhere to treatment plans for rheumatic conditions;
- Investigated obstacles to efficient treatment, such as healthcare inequalities and resource constraints.

In order to verify that the selected studies were in accordance with the study goals, they were subjected to a comprehensive examination.

#### 2.3.4. Limitations of the Study

Although this procedure was intended to be thorough, some restrictions must be acknowledged:

- Exclusion of gray literature: Unindexed but important research, such as institutional reports and conference papers, may have been omitted.
- Language limitations: Only studies published in English were included, which might exclude important work in other languages.
- Limitations on database selection—Restricting searches to specific platforms (PubMed, Scopus, Web of Science, etc.) may have resulted in excluding studies from other sources.
- Restricting the publication period (2014–2025)—While this guarantees the inclusion of current and relevant studies, it may have left out earlier fundamental research that may have offered historical context for the development of rheumatology healthcare treatments.

#### 2.3.5. Screening Process and Inclusion Decisions

To ascertain relevance and methodological rigor, each study went through two levels of review.

During the abstract and headline screening stage, each document was evaluated by two separate reviewers to determine its relevance to the study question. At this point, studies that did not specifically address nursing interventions in treating rheumatic illnesses were omitted. Any differences between reviewers were settled through dialogue or, if necessary, by consulting a third reviewer.

Studies that passed the initial screening were subjected to thorough full-text analysis using established inclusion and exclusion criteria. Two independent reviewers examined each article, ensuring consistency and reducing bias. Disagreements in the selection of studies were discussed and solved, with input from a third reviewer as needed.

#### 2.3.6. Data Collection Methods

Data extraction process

A standardized data extraction form was developed in Microsoft Excel, ensuring a consistent approach to collecting information from all included studies.

To maintain accuracy and reliability, data extraction was carried out by two independent reviewers. A third reviewer cross-checked the extracted data for completeness and correctness, resolving any discrepancies that arose.

Extracted data included details of study characteristics such as author, year, study design, sample size, and setting.

Specific nurse interventions were documented, covering areas such as pain management, functional rehabilitation, patient education and multidisciplinary care. Primary outcomes assessed included pain reduction, functional improvement, quality of life and adherence to treatment plans.

This rigorous and systematic approach ensured that study selection and data collection processes were transparent, reproducible, and methodologically sound.

## 3. Results

### 3.1. Overview of Selected Studies

A comprehensive and systematic search of seven major databases was meticulously carried out, producing a substantial pool of 101,220 results. This meticulous approach ensures the inclusivity of the review process and instills confidence in the thoroughness of our research.

After eliminating duplicate records (20,000) and studies excluded for reasons such as lack of relevance, incomplete data, or methodological limitations (80,502), 718 articles remained for selection.

During the initial screening phase, 525 records were meticulously excluded based on predefined criteria, primarily because they did not focus on medical care in rheumatic diseases. In the first phase of review, 193 studies were assessed for eligibility, with articles meticulously assessed for their relevance to nursing interventions (pain management, functional rehabilitation, patient education), multidisciplinary care (collaboration between nurses, rheumatologists, and therapists), patient adherence and self-care strategies in the management of rheumatic conditions.

In the second phase, the authors conducted a detailed assessment by applying stricter inclusion criteria concerning the effectiveness of healthcare interventions, their impact on patients’ quality of life, and barriers to accessing healthcare. We excluded studies that focused solely on pharmacologic treatments or lacked a thorough analysis of healthcare strategies.

The final selection, carefully curated from a large pool of studies, included 86 publications. These studies, published between 2014 and 2025, offer significant and valuable insights into various aspects of healthcare for rheumatic diseases. This meticulous selection process ensures the quality and relevance of the studies included in our review.

Key findings highlight the effectiveness of nurse-led interventions in pain management and functional recovery. Patient education is crucial for improving treatment adherence and disease management. This intervention empowers patients with knowledge and skills to manage their condition effectively, enhancing their quality of life.

Additionally, the importance of multidisciplinary collaboration in improving patient outcomes and the challenges and barriers to providing optimal care for patients with rheumatic diseases are emphasized.

These findings contribute to a comprehensive understanding of how nursing strategies influence the management and progression of rheumatic diseases. By integrating data from multiple medical disciplines, this approach offers insight into the impact of nursing care on patient outcomes, functional rehabilitation, and multidisciplinary collaboration.

The studies have been systematically reviewed and synthesized to assess the effectiveness of nursing interventions, patient adherence, and challenges within nursing care. They provide objective data and clinically relevant insights that deepen our understanding of the nursing process in managing rheumatic diseases.

### 3.2. Types of Nursing Interventions

Nursing interventions for patients with rheumatic diseases are diverse and multifaceted. They address the disease’s physical symptoms of disease and focus on the educational, functional, and psychosocial aspects. These interventions include educating patients about disease management and medication adherence, supporting self-care strategies, coordinating multidisciplinary care, and monitoring patient outcomes. They are based upon clinical experience and a growing body of evidence, making them an essential part of the patient’s treatment plan.

Table 1 presents the main types of health care interventions identified in the included studies, grouped according to the intervention objective and supported by key evidence.

Pain is at the heart of health care, particularly in rheumatic diseases, where chronic pain is a major cause of disability and reduced quality of life. Nurses have a key role in pain assessment and management by frequently assessing pain intensity using validated instruments, monitoring the effectiveness of treatment, and informing patients about pharmacological and non-pharmacological strategies such as exercise, relaxation methods, and cognitive-behavioral techniques. Effective pain control not only improves physical performance but also emotional state and compliance with therapy [81]. Pharmacologic approaches include the administration of non-steroidal anti-inflammatory drugs (NSAIDs), corticosteroids, and disease-modifying anti-rheumatic drugs (DMARDs) to reduce inflammation and prevent joint damage [82,83]. However, concerns about long-term side effects such as gastrointestinal complications, osteoporosis, and immunosuppression require the integration of non-pharmacological methods [84,85]. These include heat and cold applications, paraffin therapy, acupuncture, and transcutaneous electrical nerve stimulation (TENS), which have demonstrated efficacy in reducing pain, stiffness, and improving the effects of medications [86,87].

The second major area is functional rehabilitation, which includes structured physiotherapy programs, guided exercise routines, and occupational therapy strategies aimed at maintaining joint mobility, increasing muscle strength, and preventing contractures or further disability [88,89]. Low-impact activities such as swimming, yoga and adapted physiotherapy are commonly recommended for patients with spondyloarthritis and osteoarthritis. The use of assistive devices, such as orthotics, orthotic insoles and ergonomic tools, further supports patients in daily tasks by reducing pressure on inflamed or weakened joints [90].

Patient education is another basic medical intervention. This focuses on self-management training, which includes instruction on medication adherence, symptom tracking, disease flare-ups, and lifestyle modifications [91]. Educational efforts are often provided through structured workshops, one-on-one consultations, or digital platforms such as mobile health apps for disease monitoring and goal setting [92]. In addition, nutritional counseling plays a supportive role in reducing systemic inflammation and weight management, particularly in rheumatoid arthritis and osteoarthritis. Evidence suggests that anti-inflammatory diets, including the Mediterranean diet and omega-3 fatty acid supplementation, may contribute to reduced disease activity and improved patient-reported outcomes [93].

The psychosocial support component of health care addresses the psychological burden of living with a chronic, often progressive illness. Counseling by nurses, referrals to mental health professionals, and support groups are commonly used strategies to improve emotional resilience and reduce the prevalence of depression and anxiety among rheumatic patients [94]. These interventions are particularly important as emotional distress is known to exacerbate disease activity and negatively affect adherence to treatment [95].

**Figure 1:**
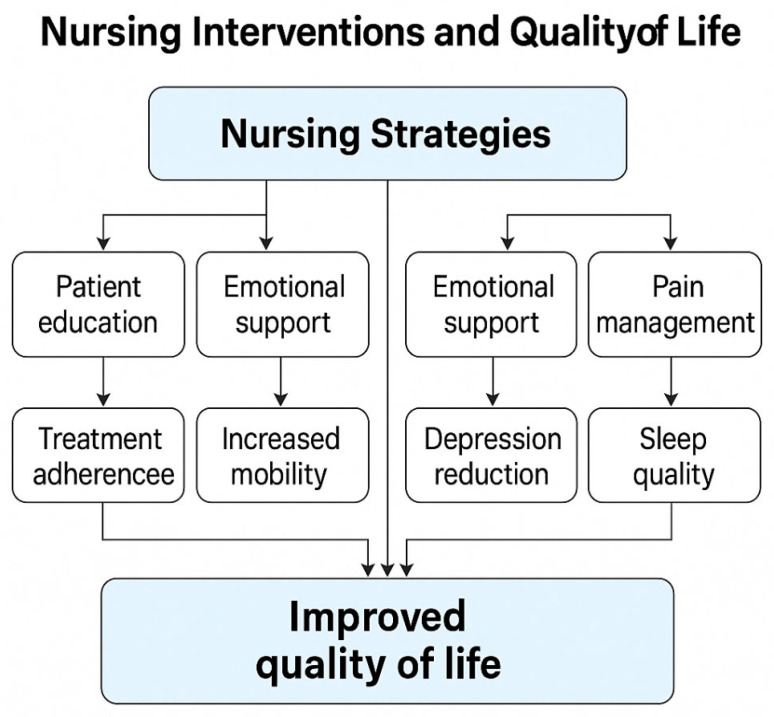
Relationship between nursing interventions and quality of life.

Overall, these various health care interventions form a multidimensional framework that supports both the physical and emotional health of people with rheumatic diseases, contributing to better long-term outcomes and improved quality of life (Figure 1).

### 3.3. Evidence of Effectiveness

Studies have consistently demonstrated the effectiveness of nurse-led interventions in improving health outcomes for patients with rheumatic diseases.

Table 2 presents the evidence on the effectiveness of nursing interventions in the care of patients with rheumatic diseases.

A systematic review by Melis et al. [96] found that tailored care for patients receiving biologic therapies resulted in better adherence to treatment and better disease management. Similarly, Alnaqbi et al. [97] highlighted the value of non-pharmacologic strategies implemented by nurses—such as supervised physical activity and lifestyle guidance—that led to improved joint function and reduced pain levels in patients with psoriatic arthritis.

Including psychological and emotional support in health care has also been linked to significant improvements in mental health outcomes. For example, cognitive-behavioral interventions provided by trained nurses helped to reduce anxiety, depressive symptoms, and fatigue in people with chronic inflammatory diseases [98]. These findings align with broader evidence on the role of exercise and patient education in alleviating disability and illness-related fatigue [99].

Nursing interventions promoting joint protection techniques and self-management education have been effective in increasing patient autonomy and adherence to treatment. According to Poh et al. [100], such educational strategies support better disease follow-up and empower patients to respond appropriately to symptom exacerbations.

A mixed-methods systematic review by Gavin et al. (2021) [101] discovered that interventions led by occupational therapists, frequently in collaboration with nurses, greatly enhanced self-management abilities in patients with rheumatoid arthritis, especially in fostering autonomy and adherence to treatment. In a similar vein, Bednarek et al. (2022) [102] highlighted that following EULAR recommendations is vital; nurse-led interventions based on these guidelines not only improved care quality but also raised patient satisfaction scores by as much as 30% in certain clinical environments. They observed differences in the consistency of implementation across institutions, which might affect results.

Incorporating rehabilitation-focused care was also demonstrated to meet the unmet needs of patients with comorbid conditions. Fedorchenko et al. (2020) [103] indicated that organized rehabilitation programs led by nurses enhanced patients’ functional independence (assessed with the Barthel Index) and decreased readmission rates by 18%. However, these programs demand considerable resource investment and cross-disciplinary collaboration, which may restrict scalability.

Valaas et al. (2019) [104] emphasized follow-up care as a key factor in adherence to self-management strategies; patients who had regular follow-up led by nurses demonstrated greater engagement and sustained long-term lifestyle changes more successfully than those lacking follow-up.

The rising adoption of digital health tools—typically implemented or overseen by nurses—has developed as an additional approach. Najm et al. (2023) [105] showed that digital interventions led by nurses, such as mobile applications and telemonitoring, resulted in better medication adherence and more regular symptom tracking. Nonetheless, gaps in digital literacy and access continue to be an issue, particularly for older individuals or those in socioeconomically disadvantaged groups.

In addition, interdisciplinary nurse-led models have demonstrated significant improvements in health-related quality of life. Auyezkhankyzy et al. [106] found that nurses’ involvement in interdisciplinary teams led to more personalized care, addressing both the physical and emotional domains of disease burden. This was echoed by Bednarek et al. [107], who emphasized that structured nurse-led consultations improve disease management and reduce patient anxiety.

Another essential but often overlooked aspect of rheumatology health care is advocacy. Nurses promote patients’ interests within interdisciplinary teams and the wider healthcare setting. This involves ensuring that referrals to rheumatologists, physiotherapists, or mental health experts are prompt, assisting with access to social services, and guiding patients in understanding insurance and disability benefits. In addition, nurses inform patients of their rights, enabling them to become involved in their care and make informed choices. In this advocacy role, nurses play a crucial role in connecting care and minimizing health inequalities, especially in marginalized communities [106].

Randomized trials, such as the ADIRA study [108], have demonstrated that nurse-guided dietary interventions can reduce inflammatory markers in rheumatoid arthritis, supporting the role of nutritional counseling in medical practice. Similarly, Raad et al. [109] confirmed that dietary adjustments combined with omega-3 supplementation—often promoted by nurses—were linked to reduced pain and stiffness.

Innovative nurse-led self-management interventions, such as the INSELMA model developed by Primdahl et al. [110], have further validated the importance of structured and interdisciplinary nursing strategies. These interventions contribute to sustained disease management, increased patient satisfaction, and improved quality of life.

Finally, the updated EULAR guidelines [111] provide strong support for an expanded role for nurses in rheumatology care. They state that nurses contribute significantly to initiating and maintaining evidence-based treatment plans with measurable effects on disease activity, patient education, and health system efficiency.

### 3.4. Comparative Analysis by Disease Type

Healthcare interventions in managing rheumatic diseases are increasingly personalized, reflecting pathophysiological complexity and clinical variability of different conditions. A comparative analysis reveals how personalized approaches enhance the effectiveness of care by addressing disease-specific problems.

Table 3 outlines disease-specific findings related to rheumatic conditions and highlights their corresponding nursing implications.

In rheumatoid polyarthritis (RA), which is characterized by chronic inflammation and joint destruction, nurses play a critical role in promoting joint protection strategies, providing education on early recognition of symptoms, and facilitating monitoring of disease-modifying treatments [112]. Recent updates of the EULAR emphasize structured patient education, support for self-management, and routine follow-up of disease activity as core responsibilities of nurses [113]. Advanced practice nurses also contribute to early flare detection and medication optimization, which are essential in preventing joint damage [114].

In osteoarthritis (OA), which usually involves mechanical degeneration of joints rather than autoimmunity, health care focuses on weight control, improving mobility, and reducing pain through physical therapy [115]. Interventions such as supervised exercise programs, ergonomic counseling, and support with assistive devices have been shown to improve stiffness and function [116]. Nurses have a key role to play in providing long-term lifestyle counseling targeting modifiable risk factors such as obesity and inactivity, particularly in older adults [117].

In systemic lupus erythematosus (SLE), a multisystem autoimmune disorder, medical care must take into account organ involvement, especially renal function, cardiovascular risk, and fatigue management [118]. Evidence supports the need for close monitoring of blood pressure, urinary protein levels, and psychosocial support, as SLE disproportionately affects women and young adults, often during peak reproductive years [119]. Nurses also provide sun protection education, adherence to immunosuppressive therapy, and reproductive counseling, which are essential in managing crises and preventing complications [120].

When addressing spondyloarthritis (SpA), particularly ankylosing spondylitis, the focus is on postural correction, spinal mobility exercises, and support for biologic therapies [121]. Nurses contribute to early detection, especially in young men with chronic back pain, and facilitate access to rheumatologic care [122].

In psoriatic arthritis, where cutaneous and joint symptoms coexist, nurses are involved in symptom management across multiple domains, often integrating dermatologic and rheumatologic protocols [123]. Education on the side effects of medications, particularly for biologic therapies, and the psychosocial burden of visible skin disease are also important aspects of care [124].

Finally, in juvenile idiopathic arthritis (JIA), which affects children and adolescents, medical care includes age-specific pain assessment, support for school integration, and coordination with pediatric specialists. Particular attention is given to family education and emotional support, as the disease often affects the entire household dynamic [125]. Nurses also advocate for transitional care planning to support adolescents as they transition to adult health services [126].

### 3.5. Multidisciplinary Collaboration

The effective management of rheumatic diseases relies heavily on multidisciplinary collaboration, with nurses playing a central coordinating role alongside rheumatologists, physiotherapists, occupational therapists, psychologists, dieticians, and social workers.

This integrated approach enables comprehensive care that extends beyond medical treatment and addresses the functional, emotional, and social dimensions of the patient’s experience [127].

In this context, nurses help to educate patients about the course of the disease, monitor responses to treatment, and encourage adherence to medication regimens. Their continuous presence in the clinical setting makes them essential in detecting early signs of disease exacerbation and facilitating timely interventions [128]. When nurses work together with rheumatologists, it becomes possible to optimize drug therapy and implement early adjustments of disease-modifying medications, reducing the likelihood of long-term complications [129].

Physiotherapists and occupational therapists are key partners in multidisciplinary teams. Their collaboration with nurses supports the restoration of mobility, functional independence, and the use of assistive devices that reduce stress on affected joints. This combined input improves patients’ commitment to rehabilitation and reduces the risk of further disability [130,131,132].

Psychologists contribute to the team by addressing the psychological burden often associated with chronic rheumatic conditions. Nurses who identify signs of emotional distress can ensure timely referral to mental health specialists. Such coordination is vital, as untreated depression or anxiety can reduce adherence to treatment and worsen disease outcomes [133,134].

Nutritional guidance also plays an important role. Dietitians and nurses work together to support anti-inflammatory eating habits, helping patients manage fatigue, weight and comorbid conditions. Patients benefit from structured, realistic meal plans that align with medical advice and cultural preferences [135].

Social workers complement the care team by assisting patients in accessing community resources, healthcare entitlements, and emotional support services. Nurses, being in close contact with patients, can alert social workers to problems related to financial issues, distress, or inadequate support at home, ensuring that patients receive comprehensive psychosocial care [136,137].

The value of this multidisciplinary collaboration has been consistently demonstrated in the literature. Patient-centered care that integrates medical, functional, and psychosocial elements is associated with improved health-related quality of life, increased satisfaction with care, and better outcomes in disease management [138,139,140].

Despite these benefits, several barriers remain. Fragmentation of services, insufficient interdisciplinary training and lack of formal structures for communication between team members can prevent collaborative care from reaching its full potential. In some settings, nurses are not always skilled or trained to take on active coordinating roles, and a shortage of specialized professionals, particularly in rural areas, may limit access to multidisciplinary support [141,142].

Emerging models of care suggest that nurse-led case management within multidisciplinary teams increases efficiency and continuity of care. These models have demonstrated reductions in hospitalizations and improvements in self-management among patients with complex rheumatic conditions [143,144]. At the same time, educational reforms and policies that promote nurse autonomy and team-based care are essential to support this development [145,146].

Continued investment in multidisciplinary collaboration, particularly in strengthening the role of nurses, is essential for the future of rheumatologic care. As the complexity of disease presentations increases, so does the need for integrated models of care that support the whole person, not just the disease [147].

### 3.6. Barriers to Implementation

Despite the proven effectiveness of healthcare interventions in the management of rheumatic diseases, their implementation in clinical practice is often constrained by multiple barriers. One of the most pressing problems is the limited access to specialized rheumatology care, particularly in rural or underserved regions. Patients in these areas frequently face delays in diagnosis and initiation of treatment, which can lead to disease progression and irreversible joint damage. This challenge is compounded by the lack of structured referral systems and poor integration between primary and specialized services [148,149].

Financial constraints are a critical obstacle for many patients, particularly in low- and middle-income countries where comprehensive insurance coverage is lacking or insufficient. The high cost of biological therapies, diagnostic imaging, and regular consultations can be prohibitive, often forcing patients to choose between essential needs and medical care [150,151]. Disparity in access to affordable care contributes to inequalities in adherence and long-term outcomes [152].

Another major barrier is the shortage of specialized rheumatology nurses. The complexity of managing chronic autoimmune conditions requires specialized knowledge, but many healthcare systems report a shortage of qualified nurses. This workforce shortage limits the scalability and consistency of care, reduces opportunities for patient education, and increases burnout of existing staff [153,154].

Healthcare fragmentation further hinders effective implementation. When communication between multidisciplinary team members is poor or disorganized, patients may experience gaps in continuity of care, conflicting advice, or duplicate services. Educational gaps among healthcare providers also contribute to inconsistent application of evidence-based care strategies, particularly in community or generalist settings [155,156].

Fear of treatment side effects and low health literacy among patients also affect adherence to healthcare-led interventions. Individuals often discontinue treatment due to concerns about long-term consequences such as weight gain, infections or gastrointestinal complications. In the absence of sustained counseling and trusting relationships with qualified nurses, many of these concerns remain unsolved, leading to premature withdrawal from treatment [157,158].

Technological and digital innovations, such as mobile health monitoring, virtual consultations, and patient empowerment apps, offer promising ways to address access and adherence challenges. However, barriers related to digital literacy, internet connectivity and system integration persist. Although telemedicine has improved continuity of care during crises such as the COVID-19 pandemic, its uptake remains uneven and cannot fully replace in-person assessment in many cases [159,160,161].

Another neglected barrier is the mental and emotional burden on both patients and their families. Chronic pain, fatigue, and disability can contribute to depression, anxiety, and social withdrawal, which in turn affect motivation and commitment to treatment plans. The lack of accessible psychological support integrated into rheumatologic care leaves a critical gap in service delivery [162,163].

Finally, institutional inertia, policy limitations, and underinvestment in medical education and care infrastructure inhibit systemic change. Without coordinated strategies to expand training programs, fund nurse-led clinics, and streamline multidisciplinary pathways, the broader impact of healthcare interventions on population health outcomes remains limited [164,165].

## 4. Discussion

The results of this synthesis highlight the central role that healthcare interventions play in the overall management of rheumatic diseases. Their impact is multifaceted, addressing physical, psychological, and social dimensions of health. However, the evidence reviewed also reveals critical complexities and contextual nuances that need to be addressed to optimize their implementation.

Non-pharmacological approaches such as joint protection, pain management through heat therapy, exercise, and patient education consistently demonstrate benefits in terms of symptom control and quality of life. However, variability in individual response and accessibility to these interventions remains substantial. Socioeconomic and geographic disparities continue to influence patients’ ability to engage in regular care, particularly in resource-limited settings where specialized care is insufficient or underfunded [166].

Patient adherence and activation levels differ significantly between disease types and care models. Individuals with chronic inflammatory arthritis often face unique psychological burdens, including isolation and reduced self-efficacy, which are exacerbated when multidisciplinary support is inconsistent or absent [167]. In addition, patients with osteoarthritis and other degenerative conditions may face a more fragmented trajectory of care, leading to increased emergency department visits that could otherwise be prevented by better outpatient support structures [168,169].

The relationship between systemic inflammation, comorbidities, and cardiovascular risks in patients with rheumatic diseases adds further complexity to the responsibilities of the nurse. Nurses are increasingly required to monitor cardiovascular health parameters and guide patients through lifestyle modification to mitigate long-term risks, often without the support of integrated cardiologic pathways [170].

Behavioral and motivational barriers are also evident. Even when nurse education is available, not all patients show adequate activation or compliance. This is particularly evident in younger patients or those with low health literacy, where passive roles in care persist despite efforts to promote self-management [171]. In addition, both patients and physicians may have conflicting expectations about the scope and effectiveness of chronic pain management, leading to dissonance in treatment goals and reduced therapeutic alliance [172].

Dietary interventions are emerging as promising adjunctive strategies, particularly anti-inflammatory diets, but current evidence remains inconsistent in terms of comparative effectiveness. More robust clinical trials are needed to establish superiority or synergism between different nutritional models and their interaction with pharmacologic regimens [173].

Digital health tools, including telemonitoring and remote coaching platforms, have demonstrated the potential to extend healthcare beyond clinical settings. However, issues of digital divide, lack of personalization, and inconsistent patient engagement remain barriers to their wider adoption [174].

Finally, the influence of socio-economic status on quality of life and medication adherence cannot be underestimated. Financial hardship, limited insurance coverage, and competing life demands often correlate with lower adherence and poorer health outcomes, underscoring the need for more equitable patterns of care and the integration of social support into health care plans [175].

In 2025, Juan Nicolás Cuenca-Zaldívar and his team established that the integration of climatic factors in the assessment and management of chronic musculoskeletal pain offers considerable opportunities for the development of personalized self-care strategies and thus contributes significantly to improving the quality of life of patients with rheumatic conditions [176].

In the light of these findings, it becomes clear that while healthcare interventions are essential, their success depends on a web of systemic, interpersonal and contextual factors. Future research and health policies need to address the heterogeneity of patient populations, strengthen multidisciplinary coordination, and prioritize accessibility to ensure that the benefits of specialized health care are universally realized.

This narrative analysis has several limitations that must be acknowledged when interpreting the findings. One of the main constraints is the exclusion of the gray literature, which could have led to the omission of relevant but unindexed data, such as conference proceedings, theses or institutional reports. Although rigorous inclusion criteria were applied, this may have introduced a publication bias, favoring studies with positive or statistically significant results.

Language restrictions are also a potential limitation, as only articles published in English were included in the review. This may have excluded valuable studies conducted in other languages, particularly from non-English speaking regions where the management of rheumatic diseases may differ due to cultural, infrastructural, or economic factors.

In addition, some of the studies lacked detailed reporting of care-specific outcomes, limiting the ability to assess the direct impact of care interventions on clinical parameters. In some cases, multidisciplinary interventions were reported without clearly distinguishing the contribution of the nursing component, complicating the attribution of outcomes.

Another limitation concerns the reliance on self-reported measures in many studies, particularly those assessing adherence to treatment, quality of life, or symptom severity. These measures are subject to reporting bias and may not accurately reflect objective clinical changes.

Finally, although this review included a broad time period and a large number of sources, it is possible that relevant studies published outside the selected period were excluded.

## 5. Conclusions

This review emphasizes the essential role of healthcare interventions in improving clinical outcomes and quality of life among patients with rheumatic diseases. In a variety of care settings, evidence suggests that tailored nursing strategies, both pharmacologic and non-pharmacologic, contribute significantly to pain relief, improved mobility, greater functional independence, and better adherence to treatment. The data highlight not only the effectiveness of individual interventions such as exercise programs, joint protection techniques, and patient education, but also the synergistic value of integrating them into long-term care plans.

The analysis shows that healthcare interventions are more effective when they are tailored to the specific type of disease and patient characteristics. Interventions for rheumatoid arthritis focus mainly on inflammation control and joint protection, while interventions for osteoarthritis target weight management and lifestyle modification. Patients with systemic lupus erythematosus benefit from multidisciplinary monitoring due to multi-organ involvement, further reinforcing the need for disease-specific care pathways.

Multidisciplinary collaboration has emerged as a central pillar of effective care. Nurses, when integrated into coordinated care teams alongside rheumatologists, physiotherapists, occupational therapists, psychologists and dieticians, are better positioned to provide holistic, patient-centered care. This interprofessional model not only improves clinical outcomes but also strengthens patient engagement, psychological well-being and continuity of care.

Despite these benefits, implementation of healthcare interventions faces persistent barriers. The review identifies systemic problems such as shortages of health workers, inequalities in access to specialized care and infrastructural disparities—particularly in rural or low-resource settings. Patient-related challenges, including low health literacy, fear of medication side effects and inconsistent adherence, further complicate care delivery.

Emerging solutions, such as the integration of digital health technologies, telemonitoring, and mobile health apps, offer promising avenues to extend the reach and responsiveness of healthcare interventions. However, their successful implementation requires careful design, user-friendly platforms, and support structures to overcome digital exclusion.

In general, the complexity of rheumatic disease care requires a multidimensional approach in which healthcare plays a strategic, adaptive, and evidence-based role. While existing research supports the efficacy of many nursing strategies, variability in study design, quality of evidence, and outcome measures highlights the need for more robust clinical trials, improved categorization of evidence, and standardization of reporting. Addressing these gaps will be essential for shaping future rheumatology nursing practice and ensuring equitable, high-quality care for all patients.

## Figures and Tables

**Table 1:** Types of Nursing Interventions.

Ref.	Study Type	Focused Intervention	Targeted Domain	Key Findings/Outcomes
[81]	Review	Non-pharmacological pain relief (e.g., TENS, cold/heat)	Pain Management	TENS, thermal therapies reduce chronic rheumatic pain; effective as an adjuvant.
[82]	Narrative review	Integrated treatment approaches	Functional Rehabilitation and Education	Highlights the importance of nurse-led interventions and self-care support.
[83]	Review	Psychological impact and affective disturbance	Psychosocial Support	Emphasizes the link between emotional distress and disease flares in RA.
[84]	Experimental	Targeted drug delivery (nanoparticles)	Pain and Disease Management	Supports precise medication interventions; nurses monitor systemic reactions.
[85]	Observational	Lupus care post-COVID	Patient Education and Monitoring	Nurses are involved in organ system monitoring and risk communication.
[86]	Systematic Review	Nursing-sensitive outcomes	Multiple Domains	Validates measurable outcomes in RA care: pain, fatigue, function.
[87]	Randomized Controlled Trial (COMEDRA)	Nurse-led comorbidity and self-monitoring	Patient Education	Demonstrates improved patient adherence and disease awareness.
[88]	Review	Safety of anti-rheumatic drugs	Pharmacological Management	Nurses play a role in monitoring adverse effects and compliance.
[89]	Clinical Guidance	Early arthritis management	Pain Management	Highlights the importance of early intervention, nurse involvement in triage.
[90]	Review	Nonpharmacological treatment in RA	Pain Management	Advocates combine TENS, exercise, and counselling.
[91]	Clinical Review	Pain management without medication	Pain and Psychosocial	Integrates CBT, relaxation, and physical therapies.
[92]	Clinical Guidelines	Sjögren’s management incl. fatigue	Pain and Psychosocial	Recommends multidisciplinary strategies; nurses lead fatigue education.
[93]	Book chapter	Chronic pain modalities	Pain Management	Presents evidence on massage, manual therapies in chronic pain.
[94]	Review	Psycho-neuro-immunological approach	Psychosocial and Functional	Suggests holistic nurse-led care improves immune balance.
[95]	Experimental	Exercise and fatigue in rheumatics	Functional Rehabilitation	Demonstrates that exercise reduces fatigue and improves mobility.

**Table 2:** Evidence on the effectiveness of healthcare interventions for rheumatic disease patient care.

Ref.	Study Type	Focus of Intervention	Key Findings	Implications for Nursing Practice
[96]	Systematic Review	Nursing interventions for patients on biologics	Improved adherence and disease control	Supports nurse involvement in biologic therapy management
[97]	Expert Consensus	Non-pharmacological care for Psoriatic Arthritis	Enhanced physical function, reduced symptoms	Nurses should promote physical therapy and patient counselling
[98]	Review	Non-pharmacological, non-surgical OA treatment	Significant symptom relief through conservative care	Validates nursing-led interventions for OA
[99]	Randomized Controlled Trial	Joint protection and physical activity training	Increased self-efficacy and mobility	Encourages an active nurse role in RA education and rehabilitation
[100]	Integrative Review	RA patient experiences	Identified educational and emotional needs	Highlights personalised education by nurses
[101]	Mixed-Methods Systematic Review	Occupational therapy impact in RA	Boosted self-management and daily function	Advocates nurse-OT collaboration for holistic care
[102]	Narrative Review	EULAR-based nursing roles	Aligned care improves patient outcomes	Reinforces evidence-based nursing practice standards
[103]	Observational Study	Rehab needs in inflammatory rheumatic diseases	Notable gaps in nursing-led rehabilitation	Nurses need to expand rehab service coordination
[104]	Multicentre Cohort	Follow-up adherence in musculoskeletal rehab	Positive link between follow-up and self-management	Underlines the importance of sustained nursing support
[105]	Systematic Review	Mobile health apps for self-management	Digital tools enhance treatment adherence	Nurses should integrate mHealth tools in care plans
[106]	Cross-sectional Study	Nurse roles in disease management	Broad scope: education, monitoring, advocacy	Calls for expanded training and autonomy for nurses
[107]	Literature Review	Implementation of EULAR recommendations	Effective disease monitoring and counselling	Confirms the necessity of guideline-informed nursing
[108]	Randomized Controlled Trial (Crossover)	Anti-inflammatory diet in RA	Lowered disease activity with dietary support	Validates nurse-led nutrition interventions
[109]	Systematic Review	Diet + omega-3 in RA	Moderate pain and stiffness reduction	Supports the inclusion of dietitians in nursing care models
[110]	Intervention Development Study	INSELMA: Interdisciplinary nurse-led model	Improved engagement and self-management	Showcases a model for advanced nurse coordination
[111]	EULAR Guideline Update	Nursing in chronic arthritis management	Clear role definitions and practice standards	Foundation for structured, effective nursing roles

**Table 3:** Disease-Specific Findings and Nursing Implications in Rheumatic Conditions.

Study (Citation)	Disease Focus	Main Findings	Nursing Intervention Implications
[112]	General RMDs	Emphasises adherence as a core challenge in chronic rheumatic care	Nurses play a key role in adherence counselling, screening for non-compliance, and promoting follow-up routines
[113]	SystemicLupus Erythematosus (SLE)	Multidisciplinary care improves outcomes and quality of life in SLE	Nurses coordinate interdisciplinary teams, monitor multi-organ complications, and support fatigue and mental health
[114]	Osteoarthritis (OA)	Nurse-led CBT significantly reduced pain and improved coping	Nurses can deliver behavioral pain management programs to improve QoL
[115]	Inflammatory arthritis	Strong support for structured patient education in IA	Nurses are central in educational planning, empowering patients to self-manage and monitor disease activity
[116]	Inflammatory arthritis	Psychological factors influence disease burden	Nursing support should include emotional screening and tailored mental health referrals
[117]	Autoimmune rheumatic diseases	PTSD and trauma are linked to flare-ups and disease severity	Nurses should assess trauma history and integrate stress-reduction interventions
[118]	Rheumatoid arthritis (ROA), Osteoarthritis, Systemic Lupus Erythematosus	National data confirm a high burden on mental and physical health	Nurses must adopt holistic strategies, addressing function, fatigue, and mental well-being
[119]	Inflammatory arthritis	Rehabilitation is often underused despite evidence	Nurses can advocate for rehab referrals, deliver mobility training
[120]	General rheumatology	Nurses are essential in outpatient care coordination and monitoring	Supports nurse-led clinics, continuous patient follow-up, and care continuity
[121,122]	Rheumatoid arthritis, Inflammatory Bowel Disease (IBD)	Diet impacts inflammatory markers and energy levels	Nurses assist in nutritional planning and dietary education tailored to comorbidities
[123]	General RMDs	Effective self-management support improves engagement	Nurses should use structured self-management protocols
[124]	Chronic complex needs	Effective coordination improves care for patients with comorbidities	Nurses act as care navigators, ensuring integration between rheumatology, primary care, and rehab
[125]	Chronic diseases	Regular nurse-led support boosts treatment adherence	Validates the use of nurse-led self-management clinics in routine care
[126]	General nursing	Workload limits access to continuous development Trends show an increase in DMARDs and biologics	Overburdened nurses may lack resources to stay updated, highlighting the need for professional development support Nurses support medication monitoring, educate patients on biologic safety, and adherence

## Data Availability

No new data were created or analyzed in this study.

## Abbreviations

The following abbreviations are used in this manuscript:

ACPA	anti-citrullinated protein antibodies
APS	antiphospholipid syndrome
AS	atherosclerosis
DMARD	disease-modifying antirheumatic drugs
bDMARDs	biologic disease-modifying antirheumatic drugs
CAD	coronary artery disease
CRP	C-reactive protein
CVD	cardiovascular disease
DM	diabetes mellitus
EBV	Epstein-Barr virus
EULAR	European Alliance of Associations for Rheumatology
FLS	fibroblast-like synoviocytes
HDL	high-density lipoprotein
ICAM-1	intercellular adhesion molecule-1
IL-1	interleukin-1
IL-1β	interleukin-1 beta
IL-6	interleukin-6
LDL	low-density lipoprotein
MMPs	matrix metalloproteinases
MRI	magnetic resonance imaging
NF-κB	kappa-light-chain-enhancer of activated B cells
NSAIDs	nonsteroidal anti-inflammatory drugs
OA	osteoarthritis
RA	rheumatoid arthritis
RF	rheumatoid factor
SLE	systemic lupus erythematosus
TENS	transcutaneous electrical nerve stimulation
TNF-α	factor-alpha
VCAM-1	vascular cell adhesion molecule-1
WHO	World Health Organization
